# Therapeutic Potential of Ozonated *Ocimum basilicum* L. from Saudi Arabia: Phytochemical Characterization and Enhanced Bioactivities

**DOI:** 10.3390/ph18081223

**Published:** 2025-08-19

**Authors:** Husam Qanash, Sulaiman A. Alsalamah, Abdulrahman S. Bazaid, Mohammed Ibrahim Alghonaim, Amro Duhduh, Ibtisam Hudani

**Affiliations:** 1 Department of Medical Laboratory Science, College of Applied Medical Sciences, University of Ha’il, Hail 55476, Saudi Arabia; 2 Medical and Diagnostic Research Center, University of Ha’il, Hail 55473, Saudi Arabia; 3 Department of Biology, College of Science, Imam Mohammad Ibn Saud Islamic University (IMSIU), Riyadh 11623, Saudi Arabia; 4 Department of Medical Laboratory Technology, College of Nursing and Health Sciences, Jazan University, Jazan 45142, Saudi Arabia; 5 Pharmacy Department, Jazan University Hospital, Jazan University, Jazan 45142, Saudi Arabia

**Keywords:** medicinal plants, biological activities, polyphenols, flavonoids, cytotoxicity

## Abstract

**Background/Objectives**: Medicinal plants are an abundant source of bioactive molecules, particularly in arid environments, such as Saudi Arabia, where *Ocimum basilicum* L. (Saudi basil) has long been used for its therapeutic properties. This study aimed to examine the phytochemical profile and bioactivities of non-ozonated (untreated) and ozonated methanolic extracts of *O. basilicum* and to determine whether ozonation enhances their biological effects, with a focus on antidiabetic, anti-Alzheimer, anti-inflammatory, antimicrobial, and cytotoxic properties. **Methods**: Fresh leaves of *O. basilicum* were extracted with methanol, subjected to ozonation, and analyzed by HPLC. In vitro assays were conducted to evaluate α-amylase, α-glucosidase, and BChE inhibition, RBC membrane stabilization, antibacterial activity against *Helicobacter pylori* and cytotoxicity using normal lung fibroblasts (WI-38) and human colorectal adenocarcinoma cell line (Caco-2). **Results**: Ozonation modified the phytochemical profile, enriching chlorogenic and rosmarinic acids. Ozonated extracts exhibited stronger inhibition of α-amylase with an IC_50_ of 5.09 µg/mL compared to 13.6 µg/mL of untreated Saudi basil and α-glucosidase (IC_50_ 6.15 µg/mL vs. 9.42 µg/mL). They also showed enhanced BChE inhibition with an IC_50_ of 13.4 µg/mL compared to 31.8 µg/mL of non-ozonated extract. In addition, ozonated extracts produced significant anti-inflammatory effects by stabilizing RBCs, with an IC_50_ of 8.04 µg/mL compared to 8.44 µg/mL for untreated extracts and 4.41 µg/mL for indomethacin. Ozonated extracts produced larger *H. pylori* inhibition zones (26.7 mm) and an MBC/MIC ratio of 1. Cytotoxicity testing revealed that ozonated extracts were less toxic to WI-38 cells, with IC_50_ values of 437.89 µg/mL versus 191.06 µg/mL, and 149.14 µg/mL compared to 103.7 µg/mL of untreated Saudi basil in Caco-2 cells. **Conclusions**: Ozonation enriches the phytochemical composition of *O. basilicum*, enhancing antidiabetic, neuroprotective, anti-inflammatory, and antibacterial activities while reducing cytotoxicity on normal cells. These findings support the potential of ozonated *O. basilicum* as a safe and promising natural therapeutic candidate for metabolic, neurodegenerative, and infectious diseases.

## 1. Introduction

Medicinal plants are widely recognized for their diverse therapeutic properties, particularly those that grow in arid and mountainous environments, where harsh climatic conditions promote the accumulation of bioactive compounds. The Kingdom of Saudi Arabia, with its vast desert ecosystems, harbors a rich diversity of wild medicinal plants that have long been used in traditional medicine and represent an underexploited resource for the development of safe and effective pharmaceuticals. Optimizing the utilization of these plants is crucial to advancing drug discovery and manufacturing in the region.

Among these species, sweet basil (*Ocimum basilicum* L.) is particularly notable for its adaptability to a wide range of temperatures and geographical regions, which has contributed to its global distribution, particularly in tropical and subtropical areas [1]. Saudi Arabia is rich in medicinal plants belonging to the Lamiaceae family, which includes *O. basilicum* L., commonly known as sweet basil or Saudi basil due to its wide distribution in the region. This aromatic plant contains an essential oil composed of nearly 200 constituents. *O. basilicum* is also native to Africa, where it is known as scent leaf, and is characterized as an annual or perennial herb. The genus *Ocimum* comprises between 50 and 150 species distributed across Africa, tropical Asia, and the Americas [2].

*O. basilicum* is rich in phytochemicals such as flavonoids, polyphenols, and tannins, which underline its broad spectrum of pharmacological activities. Documented biological effects include anticancer, anti-dyspeptic, anti-inflammatory, antioxidant, antiulcer, antiviral, anthelmintic, and wound-healing properties. Furthermore, *O. basilicum* has been reported to exert cardiovascular benefits, including hypoglycemic, hypolipidemic, and anticoagulant effects through inhibition of platelet aggregation. Traditionally, its flowers and leaves have been used as antispasmodics, digestive tonics, galactagogues, and stomach tonics. They are also applied for the management of gastroenteritis, fever, nausea, insomnia, depression, migraine, gonorrhea, dysentery, chronic diarrhea, acne, anosmia, insect and snake bites, and skin infections [3]. In Indonesia, basil leaves are widely used to treat malaria, rheumatism, hypercholesterolemia, hypertension, headache, and stroke, and are also employed as an anthelmintic [4,5,6].

The therapeutic potential of *O. basilicum* is primarily attributed to its bioactive components, including linalool, eugenol, geraniol, methyl eugenol, and 1,8-cineole [7]. These constituents contribute to its antimicrobial and antidiabetic effects, with linalool and eugenol specifically shown to inhibit biofilm formation by multidrug-resistant *Staphylococcus aureus* [8,9]. The plant has a long history of use in traditional medicine across Asia and the Pacific, particularly in the treatment of flatulence, gastric ulcers, cold sores, and tuberculosis [10,11,12]. Recently, in silico studies have demonstrated that *O. basilicum* exhibits antiviral activity against SARS-CoV-2 through strong binding affinities of apigenin-7-glucuronide (−8.77 kcal/mol) and dihydrokaempferol-3-glucoside (−8.96 kcal/mol) to viral targets [13], suggesting a potential preventive effect against COVID-19 [14]. Methanolic extracts of basil leaves have also shown antibacterial effects against *S. aureus*, *Bacillus cereus*, *Escherichia coli*, *Pseudomonas aeruginosa*, and *Vibrio cholerae* [15,16], as well as antifungal activity against *Trichoderma viride*, several *Aspergillus* species (*A. ochraceus*, *A. versicolor*, *A. fumigatus*, *A. niger*), and *Penicillium* species (*P. funiculosum*, *P. ochrochloron*) [17].

Ozone (O_3_), a triatomic form of oxygen, is one of the most powerful natural oxidizing agents with potent antibacterial, antifungal, and antiviral properties. It is widely applied in water purification, wound healing, the treatment of diabetic foot ulcers, and infections caused by methicillin-resistant *S. aureus* (MRSA) [18]. In dentistry, ozone therapy is used for both preventive and restorative purposes, as it selectively eliminates pathogenic microorganisms while preserving normal microflora and rapidly degrades organic compounds without inducing cellular toxicity [19].

Ozonation can have a substantial effect on the biological activity and extraction of bioactive substances, either favorably or unfavorably, depending on the specific plant or substance being treated, the ozone concentration, and the technique used. Due to its strong oxidizing properties, ozone can act as an elicitor, stimulating the synthesis of beneficial compounds, such as antioxidants and phenolics, while potentially influencing enzymatic browning and overall quality [20]. Ozone also has the ability to degrade mycotoxins, inactivate bacteria, and alter the type or concentration of beneficial compounds present in extracts. These effects are mediated through various chemical processes, including direct oxidation and the generation of reactive oxygen species (ROS), such as hydroxyl radicals [21].

Building on these properties, the present study investigates the biological activities of non-ozonated and ozonated extracts of *O. basilicum* L. (Saudi basil) with the aim of enhancing its therapeutic potential. This approach seeks to explore the synergistic effects of ozonation and plant bioactivity to develop novel, natural, and effective treatments for a broad range of diseases.

## 2. Results and Discussion

Medicinal plants have long been recognized as a rich source of bioactive compounds that offer therapeutic benefits with minimal side effects, and they continue to play a crucial role in modern drug discovery. *O. basilicum* L. (basil), widely distributed in the Arabian Gulf region and particularly abundant in the Kingdom of Saudi Arabia, where it is commonly referred to as “Saudi basil”, is one such plant known for its diverse pharmacological properties. In this study, wild *O. basilicum* L. samples collected from different regions of Saudi Arabia were processed for methanolic extraction, and the resulting extracts were systematically evaluated for their biological activities. The following sections present and discuss the phytochemical composition and the observed therapeutic activities of non-ozonated (untreated) and ozonated extracts, including antidiabetic, anti-Alzheimer, anti-inflammatory, antimicrobial, and cytotoxicity profiles.

### 2.1. Phytochemical Profiling of Non-Ozonated and Ozonated O. basilicum L. Extracts by HPLC

**Figure 1:**
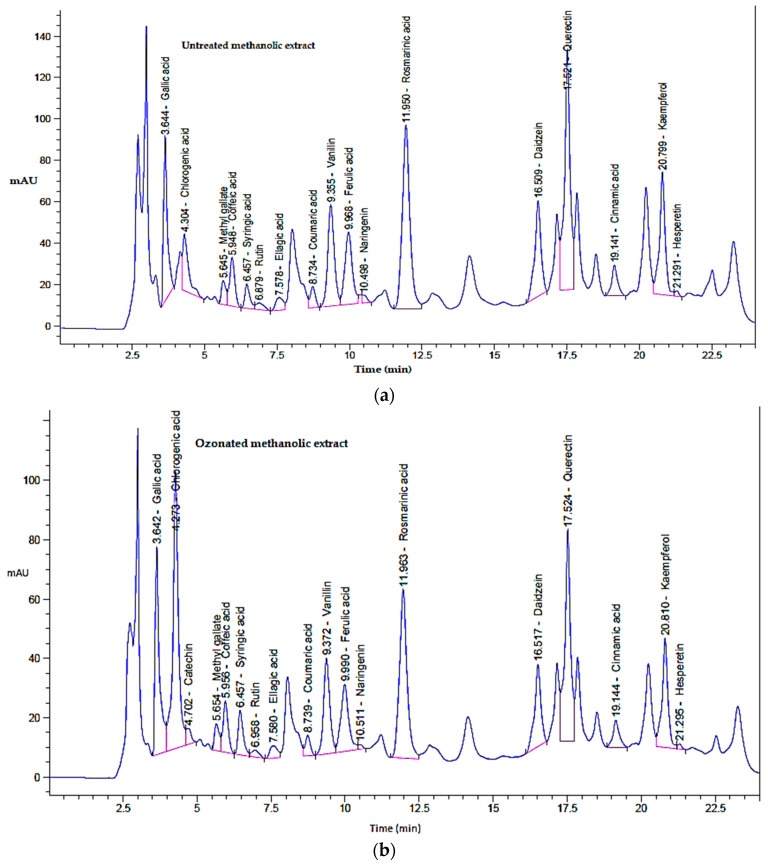
HPLC analysis of (**a**) non-ozonated (untreated) methanolic extract and (**b**) ozonated methanolic extract of *O. basilicum* L. (Saudi basil).

Ozone gas was produced using an electric boundary shockwave plasma device. A 1.9 L Drechsel cylinder containing 1.0 mg of *O. basilicum* extract was submerged in a chilling bath set at −9 °C at the output of the plasma reactor. Ozone was bubbled through the *O. basilicum* extract for 10 to 40 min at a mean rate of 1.2 mg/L/h. After ozonation, the *O. basilicum* extract was removed from the Drechsel vessel for subsequent use [22]. The chemical composition of non-ozonated (untreated) and ozonated methanolic extracts of *O. basilicum* L. was analyzed using high-performance liquid chromatography (HPLC) (Figure 1). Both extracts contained 18 major phytochemical constituents at varying concentrations (Table 1). These components are widely recognized for their therapeutic effects and have been reported to play important roles in the prevention and treatment of various diseases.

In the standard mixture of polyphenols, the identified compounds included gallic acid, chlorogenic acid, catechin, methyl gallate, caffeic acid, syringic acid, rutin, ellagic acid, coumaric acid, vanillin, ferulic acid, naringenin, rosmarinic acid, daidzein, quercetin, cinnamic acid, kaempferol, and hesperetin. Among these, coumaric acid, ellagic acid, rutin, and rosmarinic acid were among the predominant constituents.

Untreated methanolic extracts showed a total peak area of 8337.21 mAU*s. The most abundant components were rosmarinic acid (19.25%), quercetin (19.07%), kaempferol (9.73%), vanillin (9.23%), gallic acid (8.73%) and daidzein (8.30%). Catechin was absent in the untreated extract.

In contrast, ozonated methanolic extracts showed a total peak area of 6624.50 mAU*s, with a higher relative abundance of chlorogenic acid (18.23%), rosmarinic acid (15.33%), quercetin (14.58%), gallic acid (10.93%), and kaempferol (7.61%). Notably, ozonation appeared to enhance the proportion of certain phenolic acids (e.g., chlorogenic acid and catechin) (*p* ≤ 0.05) while reducing others, such as rosmarinic acids and vanillin. These compositional changes may explain the enhanced biological activity of ozonated extracts compared to untreated extracts.

These findings are consistent with previous studies. Abhishek et al. [23] reported that *O. basilicum* L. contains high levels of bioactive compounds, including polyphenols, tannins, and flavonoids. Quantitative analyses of ethanolic extracts showed total polyphenols, tannins, and flavonoids of 5.02 ± 0.06 µg gallic acid equivalent/mg, 7.80 ± 0.05 µg gallic acid equivalent/mg, and 6.00 ± 0.06 µg quercetin equivalent/mg, respectively. Similarly, Güez et al. [24] confirmed the predominance of quercetin derivatives in basil leaves. The amounts of these phytochemicals vary considerably depending on biotic and abiotic factors. For example, Aburigal et al. [25] reported that the phenolic content in *O. basilicum* grown at different locations ranged from 2086 to 25,593 mg GAE/100 g dry weight. These phytochemicals, especially rosmarinic acid, chlorogenic acid, and quercetin, are considered key therapeutic agents because of their antimicrobial, antioxidant, and anti-inflammatory activities.

The observed differences between untreated and ozonated extracts demonstrate that ozonation modifies the phytochemical profile of *O. basilicum* L., possibly enriching phenolic compounds with higher therapeutic relevance, which correlates with the improved biological activities presented in the subsequent sections.

### 2.2. Antidiabetic Activity: Inhibition of α-Amylase and α-Glucosidase

The antidiabetic activity of *O. basilicum* L. extracts was evaluated based on their ability to inhibit key starch-degrading enzymes, α-amylase and α-glucosidase, which are responsible for releasing glucose as the final product of starch hydrolysis. Inhibition of these enzymes is a well-established therapeutic strategy for controlling postprandial hyperglycemia in type 2 diabetes.

Both non-ozonated (untreated) and ozonated extracts of Saudi basil demonstrated inhibitory effects on α-amylase when compared with the standard drug acarbose (Table 2). The mean absorbance at 540 nm was recorded for all treatments at comparable concentrations. Although the untreated extract exhibited consistent α-amylase inhibition across all concentrations, the ozonated extract showed significantly higher (*p* ≤ 0.05) activity. The IC_50_ values for α-amylase inhibition were 13.6 μg/mL for untreated Saudi basil and 5.09 μg/mL for ozonated Saudi basil, compared to 3.47 μg/mL for acarbose. These findings indicate that ozonation enhances the antidiabetic potential of extract.

These results are in agreement with earlier studies. Chinelo et al. [26] reported that extracts of the aerial parts of *O. basilicum* L. significantly lowered fasting blood glucose in diabetic rats by 59.21% and 38.67% after treatment with 100 mg/kg and 200 mg/kg, respectively. Similarly, Siba et al. [27] confirmed that inhibition of α-amylase and α-glucosidase is one of the most direct approaches for controlling type 2 diabetes, and methanolic extracts of *O. basilicum* demonstrated both in vitro and in vivo activity, with 500 μg/mL of extract causing 70.2% ± 8.6 inhibition of α-glucosidase and 25.4% ± 3.3 inhibition of α-amylase.

**Figure 2:**
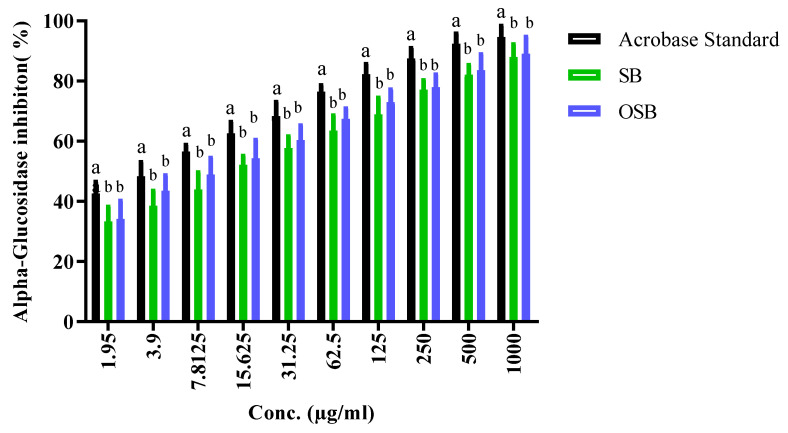
Bar graphs for statistical comparisons among SB, OSB, and the standard (data are presented as means ± SD; different lowercase letters above the columns indicate significant differences where *p* ≤ 0.05).

The Food and Drug Administration (FDA) and the Food and Agriculture Organization (FAO) have registered *O. basilicum* L. as a potent antidiabetic herb, due to its ability to inhibit intestinal digestive enzymes, including α-amylase and α-glucosidase. However, α-glucosidase is more sensitive to inhibition than α-amylase, because α-amylase initiates the hydrolysis of polysaccharides at bonds of 1,4-glycosidic resulting in the formation of disaccharides, whereas α-glucosidase hydrolyzes disaccharides to monosaccharides [28]. In this study, therefore, the inhibitory activity of the extracts on α-glucosidase was also assessed and compared with acarbose as a reference drug (Figure 2 and Table 3). Similarly to α-amylase inhibition, ozonated extracts exhibited a stronger inhibitory effect on α-glucosidase than non-ozonated (untreated) extracts. The IC_50_ values for α-glucosidase inhibition were 9.42 ± 0.6 μg/mL (untreated) and 6.15 ± 0.3 μg/mL (ozonated), compared with 2.91 ± 0.4 μg/mL for acarbose.

These findings are supported by Suresh et al. [29], who reported the presence of multiple hypoglycemic constituents in *Ocimum* species, including eugenol, caffeic acid, beta-sitosterol, chicoric acid, oleanolic acid, luteolin, ursolic acid, and rosmarinic acid. Among the genus, *O. tenuiflorum*, *O. gratissimum*, and *O. basilicum* were shown to exhibit the strongest inhibitory effects on α-glucosidase, with *O. tenuiflorum* achieving IC_50_ values comparable to acarbose.

Overall, these results demonstrate that ozonation of Saudi basil extracts enhances their antidiabetic activity, particularly through stronger inhibition of α-glucosidase and α-amylase enzymes, which is consistent with the higher abundance of phenolic compounds observed in the phytochemical analysis.

### 2.3. Anti-Alzheimer Activity: Inhibition of Butyrylcholinesterase (BChE)

BChE is a hydrolytic enzyme encoded by a distinct gene and is less widely distributed in the mammalian brain compared with acetylcholinesterase. Although it plays a limited role in classical cholinergic neurotransmission, BChE activity is known to increase in the brains of patients with Alzheimer’s disease (AD), contributing to the formation of amyloid plaques and neurofibrillary tangles—two pathological hallmarks of the disease. Inhibiting BChE has, therefore, become a promising therapeutic strategy for slowing the progression of AD by reducing plaque and tangle formation.

In this study, both non-ozonated (untreated) and ozonated extracts of Saudi basil were evaluated for their ability to inhibit BChE and were compared with rivastigmine, a standard BChE inhibitor (Table 4). At equivalent concentrations, all extracts exhibited inhibitory activity against BChE. However, ozonated Saudi basil consistently demonstrated a stronger inhibitory effect than untreated Saudi basil. The IC_50_ values for BChE inhibition were 31.8 μg/mL for untreated extracts and 13.4 μg/mL for ozonated extracts, whereas rivastigmine exhibited a much lower IC_50_ of 0.46 μg/mL.

These findings are in agreement with Atif et al. [30], who reported that both aqueous and methanolic extracts of the aerial parts of *O. basilicum* L. effectively inhibit BChE activity. The essential oil from *O. basilicum* was found to contain methyl chavicol (51.9%) and linalool (20.0%) as its main components, conferring significant antioxidant activity (16.72 ± 0.95 mmol TEs/g), ferric reducing antioxidant power (86.30 ± 2.80 FRAP mg TEs/g), cupric reducing antioxidant capacity (115.31 ± 2.03 CUPRAC mg TEs/g), and metal chelating ability (21.08 ± 3.45 mg EDTA equivalent/g). The same oil demonstrated strong BChE inhibition (1.19 ± 0.13 mg GALAEs/g).

Collectively, these results confirm that *O. basilicum* extracts, particularly after ozonation, have substantial potential as natural agents for inhibiting BChE. This supports the concept that modulation of BChE activity can be an effective therapeutic approach for reducing neurofibrillary tangle and amyloid plaque formation in Alzheimer’s disease [31,32].

### 2.4. Anti-Inflammatory Activity: Inhibition of Hypotonic Red Blood Cell Hemolysis

Hemolysis of red blood cells (RBCs) leads to the release of large amounts of damage-associated molecular patterns (DAMPs) into circulation, which in turn activate multiple inflammatory pathways. Therefore, the anti-inflammatory potential of a compound can be assessed by its ability to stabilize RBC membranes and inhibit hemolysis under hypotonic conditions.

In this study, both non-ozonated (untreated) and ozonated extracts of Saudi basil were tested for their ability to inhibit hypotonic red blood cell hemolysis (HRBC) and compared to indomethacin, a standard anti-inflammatory drug (Table 5). The absorbance at 540 nm was recorded at equivalent concentrations for all test samples. Both extracts demonstrated significant hemolysis inhibition, with the ozonated extract showing slightly greater activity than the untreated extract. The IC_50_ values for HRBC inhibition were 8.44 μg/mL for untreated Saudi basil and 8.04 μg/mL for ozonated Saudi basil, compared to 4.41 μg/mL for indomethacin.

These findings are supported by Abhishek et al. [23], who reported that ethanolic extracts of *O. basilicum* L. exhibited a stronger anti-inflammatory effect than diclofenac sodium, particularly at higher concentrations. Under hypotonic conditions, diclofenac sodium at 50 μg/mL reduced HRBC hemolysis to 64.08 ± 1.33%, while 1000 μg/mL reduced it to 39.81 ± 2.95%. In comparison, ethanolic extracts of *O. basilicum* L. reduced HRBC hemolysis to 27.18 ± 2.98% at 50 μg/mL and 1.94 ± 1.43% at 1000 μg/mL, indicating a substantially greater membrane-stabilizing effect. The protective effect of *O. basilicum* extract was 2.03 times greater than diclofenac sodium at 50 μg/mL and 1.63 times greater at 1000 μg/mL.

The membrane-stabilizing properties of *O. basilicum* extracts are attributed to their high content of phenolics and flavonoids, which are known to inhibit lysosomal enzymes (such as protein kinase C, phospholipase A_2_, and protein tyrosine kinases) and suppress inflammatory mediators including cyclooxygenase (COX), cytokines, nuclear factor kappa B, and matrix metalloproteinases [33,34]. By maintaining the stability of lysosomal and RBC membranes, these compounds prevent the cascade of cellular events that lead to tissue inflammation and damage.

Overall, these results confirm that both non-ozonated and ozonated extracts of *O. basilicum* L. exert significant anti-inflammatory effects through stabilization of RBC membranes, with ozonation providing a modest enhancement in activity.

### 2.5. Anti-Helicobacter pylori Activity

*Helicobacter pylori* (previously *Campylobacter pylori*) is a Gram-negative, helical, flagellated bacterium that colonizes the gastric mucosa. Its ability to penetrate the stomach lining and survive in acidic environments makes it a major cause of chronic gastritis, peptic ulcers, and, in severe or untreated cases, gastric carcinoma. Morphological variations from helical to rod-like or curved forms can occur due to mutations, while maintaining the bacterium’s pathogenic characteristics.

**Figure 3:**
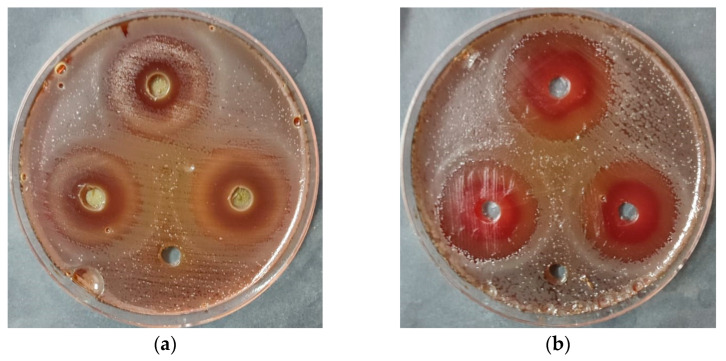
Qualitative assay of anti-*H. pylori* for (**a**) non-ozonated Saudi basil (untreated) and (**b**) ozonated Saudi basil. Well without inhibition zone loaded with DMSO.

In the present study, non-ozonated (untreated) and ozonated extracts of *O. basilicum* L. were evaluated for their antibacterial activity against *H. pylori* and compared to the standard therapy Omeclamox-Pak (amoxicillin 1000 mg, omeprazole 20 mg, and clarithromycin 500 mg) (Table 6). Qualitative assessment of antibacterial activity was performed using the agar well diffusion method, where zones of inhibition were measured around wells containing the test samples (Figure 3).

Ozonated Saudi basil demonstrated the greatest inhibitory effect, with a mean inhibition zone of 26.7 mm, which exceeded that of the standard Omeclamox-Pak control (24.5 mm), while the untreated Saudi basil extract produced an inhibition zone of 18.7 mm. The standard deviation and standard error were calculated for all experimental groups. The enhanced anti-*H. pylori* activity is likely due to bioactive compounds, such as rosmarinic acid, chlorogenic acid, catechin, quercetin, and gallic acid, all of which were identified in our HPLC analysis. These compounds were reported to exert antibacterial effects through mechanisms including disruption of bacterial membranes, inhibition of urease activity, and interference with biofilm formation [35,36,37,38].

These results align with the findings of Doha Abou Baker [39], who reported that *H. pylori* infect nearly 50% of the global population, with eradication becoming increasingly challenging due to widespread multidrug resistance. Medicinal plants, including *O. basilicum* L., have been identified as promising sources of bioactive compounds capable of suppressing *H. pylori* growth through the action of secondary metabolites. Similarly, Maria et al. [40] reported that standard therapy, based on proton pump inhibitors and antibiotics in a dual regimen, often fails due to the bacterium’s ability to acquire resistance. Recent studies suggest that ozone therapy may serve as an effective alternative; for example, a 90-day treatment protocol involving ozonated saline and oral administration of ozonated olive oil (BIOZON^®^, Orlando, FL, USA, 600 meqO_2_) resulted in a cure in 18 out of 21 patients infected with *H. pylori*.

In addition to qualitative assays, the quantitative anti-*H. pylori* activity of untreated and ozonated methanolic extracts of *O. basilicum* L. was evaluated using samples containing red blood cells (RBCs) and isotonic solutions. The results were compared with Omeclamox-Pak as a standard control (Table 7). Extracts were tested at 25%, 50%, and 75% of their minimum inhibitory concentration (MIC).

**Figure 4:**
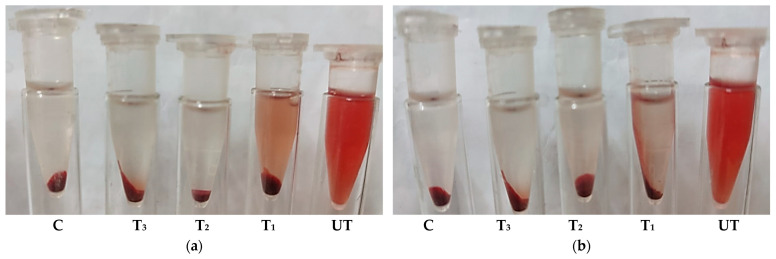
Hemolysis (%) for quantitative assay of anti-*H. pylori*: (**a**) non-ozonated Saudi basil (untreated): (C); control, (T_1_) treatment with 25% MIC, (T_2_) treatment with 50% MIC, (T_3_) treatment with 75% MIC; (**b**) ozonated Saudi basil: (C); control, (T_1_) treatment with 25% MIC, (T_2_) treatment with 50% MIC, (T_3_) treatment with 75% MIC.

In the RBC-containing samples, the ozonated Saudi basil extract showed the greatest anti-*H. pylori* activity at all concentrations (75%, 50%, and 25% MIC), followed by untreated Saudi basil and then the Omeclamox-Pak control. A similar trend was observed for hemolysis inhibition, where the lowest hemolysis occurred with the ozonated extract, indicating a superior ability to protect RBC integrity during antibacterial activity (Figure 4).

In the isotonic solution samples, ozonated Saudi basil again showed the strongest activity, particularly at 25% and 50% MIC, followed by untreated Saudi basil. However, at 75% MIC, neither untreated nor ozonated extracts demonstrated activity under isotonic conditions. These results highlight that ozonation enhances the antibacterial potency of *O. basilicum* extracts while simultaneously reducing hemolytic effects.

These findings are consistent with observations reported by Kassahun and Abebe [41], who demonstrated that *H. pylori* infection is associated with altered hematological parameters. Specifically, patients with *H. pylori* infection exhibited significantly lower mean values for hemoglobin (Hgb, *p* < 0.001), RBC count (*p* < 0.001), hematocrit (HCT, *p* < 0.001), mean corpuscular volume (MCV, *p* = 0.003), mean corpuscular hemoglobin (MCH, *p* = 0.008), and mean corpuscular hemoglobin concentration (MCHC, *p* = 0.006), whereas red cell distribution width (RDW) was significantly higher (*p* = 0.003) compared to healthy individuals. Moreover, 13.3% of patients had low hemoglobin, 7% had low RBC count, 6.4% had low hematocrit, and 18.2% had low MCV. Such hematological abnormalities in *H. pylori*-infected patients are largely attributed to chronic gastritis, iron deficiency, and vitamin B12 malabsorption caused by the infection [42,43,44,45].

Collectively, these results demonstrate that ozonated *O. basilicum* extracts not only enhance anti-*H. pylori* activity but also provide superior protection against hemolysis, which is of clinical relevance given the hematological alterations commonly associated with *H. pylori* infection.

### 2.6. Determination of Minimum Inhibitory Concentration (MIC) and Minimum Bactericidal Concentration (MBC)

The antibacterial activity of non-ozonated (untreated) and ozonated extracts of *O. basilicum* L. against *H. pylori* was further evaluated by determining their MIC and MBC in comparison with the standard therapy Omeclamox-Pak (Table 8).

For the untreated Saudi basil extract, the MIC was 15.62 μg/mL, and the MBC was 31.25 μg/mL, yielding an MBC/MIC ratio of 2, indicative of a bactericidal effect. For Omeclamox-Pak, the MIC and MBC were 31.25 μg/mL and 62.5 μg/mL, respectively, also corresponding to a bactericidal effect.

In contrast, ozonated Saudi basil exhibited equal MIC and MBC values (31.25 μg/mL), resulting in an MBC/MIC ratio of 1, which strongly confirms a bactericidal effect. These results indicate that ozonation did not compromise the bactericidal properties of the extract and suggests a more rapid bacterial killing effect.

Comparable results have been reported by Izabela et al. [46], who evaluated 26 essential oils for anti-*H. pylori* activity against the reference strain *H. pylori* ATCC 43504. Their study demonstrated MIC values ranging from 15.6 mg/L for the most potent oils (thyme, lemongrass, cedarwood, and lemon balm) to 31.3 mg/L for oregano oil, 62.5 mg/L for tea tree oil, and up to 125 mg/L for pine needle, lemon, and silver fir oils. Importantly, all tested essential oils exhibited bactericidal activity, as indicated by MBC/MIC ratios < 4.

The present findings confirm that ozonated *O. basilicum* L. extract exhibits a rapid bactericidal effect against *H. pylori*, with an MBC/MIC ratio of 1, suggesting a potential advantage over conventional therapy. Bioactive substances from medicinal plants, some of which exhibit antibacterial activity against *H. pylori*, can be extracted using polar solvents, such as water and ethanol. The type and quantity of chemical compounds extracted, as well as the extract’s efficacy against *H. pylori* and its MIC levels, can be influenced by the choice of solvent [47,48].

### 2.7. Cytotoxicity and Cell Viability in Normal Lung Fibroblasts (WI-38 Cell Line)

**Figure 5:**
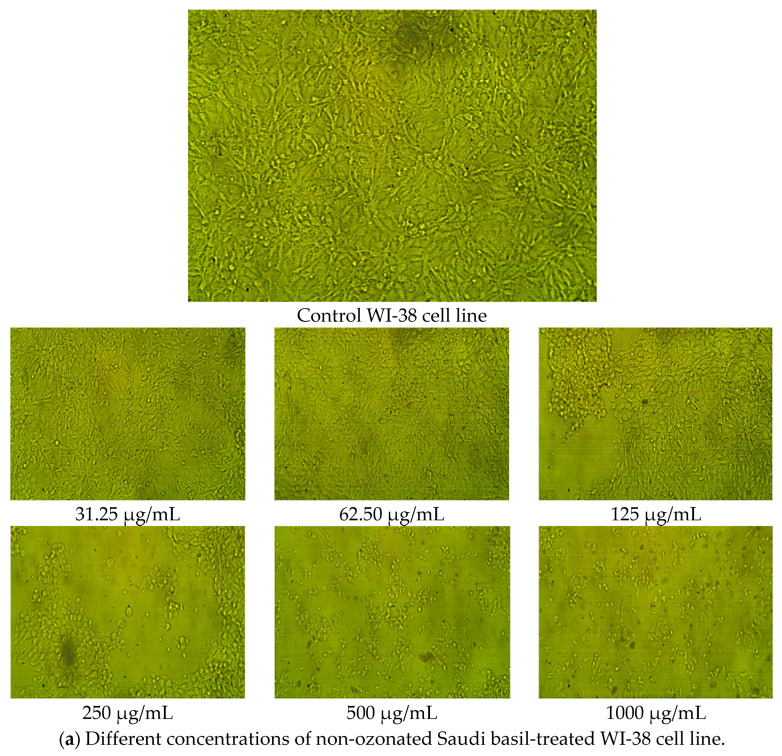

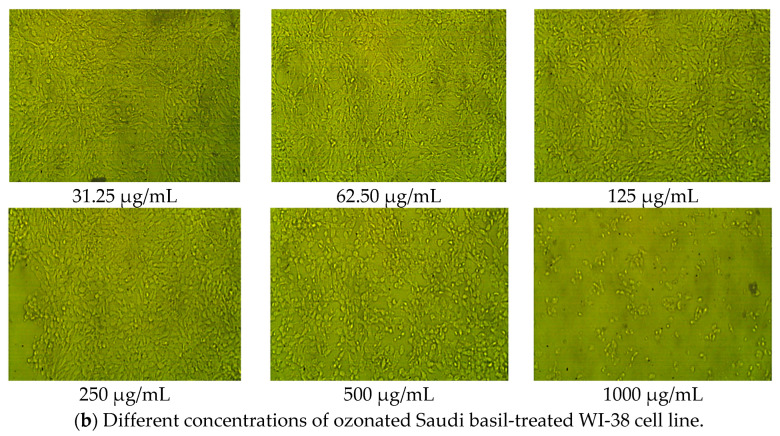
WI-38 cell line in lung tissues as a positive control, (**a**) different concentrations of ozonated Saudi basil (untreated), and (**b**) different concentrations of ozonated Saudi basil for testing cytotoxicity.

The cytotoxicity and viability effects of non-ozonated and ozonated *O. basilicum* L. extracts were assessed on WI-38 cells, a normal human lung fibroblast cell line. Morphological evaluation revealed that the WI-38 cells maintained normal appearance (no signs of shrinkage, swelling, or detachment) across all tested concentrations of both extracts (Figure 5).

Dose–response analysis (Table 9) demonstrated that non-ozonated Saudi basil (untreated) was more cytotoxic to WI-38 cells than ozonated Saudi basil, with IC_50_ values of 191.06 μg/mL and 437.89 μg/mL, respectively. At the highest tested concentration (1000 μg/mL), the untreated methanolic extract reduced cell viability by 8.35%, whereas the ozonated extract showed 95.04% toxicity, leaving 4.96% cell viability. Toxicity decreased as the concentration of both extracts was reduced, with the ozonated extract consistently showing lower cytotoxicity and higher cell viability compared with the untreated extract.

These findings are consistent with previous studies. Jegathambigai et al. [49] reported that *O. basilicum* L. extracts inhibited the proliferation of HL-60 leukemia cells, with IC_50_ values of 45.67 μg/mL and 98.1 μg/mL at 42 h of exposure. At high concentrations (100–200 μg/mL), the extracts induced cell death characterized by shrinkage and clumping. Using an MTT assay, they confirmed that *O. basilicum* extracts significantly reduced viability by 50% in HL-60 cells, demonstrating selective cytotoxic effects on cancer cells.

In the present study, however, both untreated and ozonated Saudi basil extracts exhibited low toxicity to normal WI-38 lung fibroblasts, particularly at concentrations below 200 μg/mL, with the ozonated extract being less cytotoxic. This suggests that the ozonation process enhances the therapeutic potential of the extract without increasing cytotoxic effects on normal cells.

### 2.8. Cytotoxicity and Cell Viability in Colon Epithelial Cells (Caco-2 Cell Line)

**Figure 6:**
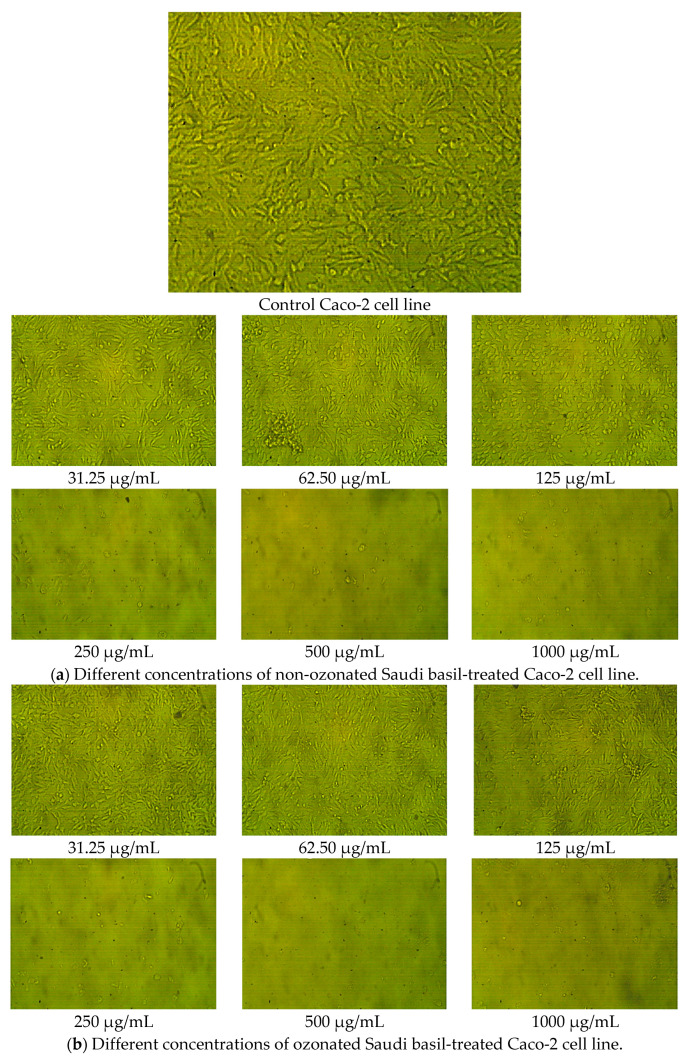
Caco-2 cell line in colon tissues as a positive control, (**a**) different concentrations of ozonated Saudi basil (untreated), and (**b**) different concentrations of ozonated Saudi basil for testing cytotoxicity.

The cytotoxic effects of non-ozonated (untreated) and ozonated *O. basilicum* L. extracts were also evaluated on the human colorectal adenocarcinoma cell line (Caco-2). Similarly to WI-38 cells, morphological assessment showed no abnormal changes, such as swelling, shrinkage, or detachment, in untreated cells across all concentrations of both extracts (Figure 6).

**Figure 7:**
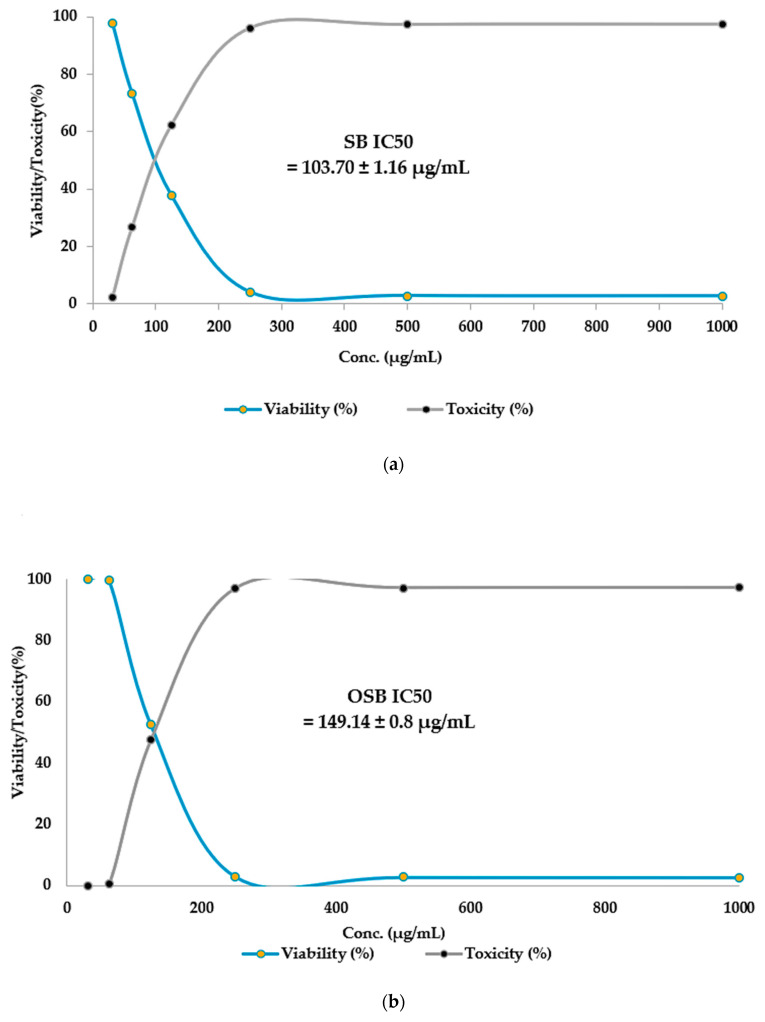
Statistical analysis using line charts for (**a**) SB and (**b**) OSB versus Caco-2 cells.

Dose-dependent cytotoxicity assays (Table 10 and Figure 7) revealed that non-ozonated Saudi basil was more toxic to Caco-2 cells than ozonated Saudi basil, with IC_50_ values of 103.7 μg/mL and 149.14 μg/mL, respectively. At the highest concentration (1000 μg/mL), the untreated extract caused 97.34% cytotoxicity (2.66% viability), whereas the ozonated extract caused 97.24% cytotoxicity (2.76% viability). Although cytotoxicity declined as concentrations decreased, the ozonated extract consistently demonstrated reduced toxicity and higher cell viability compared with the untreated extract.

These results are consistent with earlier findings. Zarlaha et al. [50] reported that ethanolic extracts of *O. basilicum* L. essential oil displayed antiproliferative activity against several human cancer cell lines, including HeLa (cervical adenocarcinoma), FemX (melanoma), K562 (chronic myelogenous leukemia), and SKOV3 (ovarian carcinoma). The activity was attributed to phytochemical constituents, such as rosmarinic acid, caffeic acid, eugenol, isoeugenol, and linalool, which have been associated with cytotoxic and antiproliferative properties. The present findings suggest that ozonated Saudi basil extracts exhibit a lower cytotoxic effect on colon epithelial cells than untreated extracts, supporting their potential therapeutic use with a better safety profile for normal tissue. A lower IC_50_ value for cancer cells (e.g., Caco-2 cells used in this study) is generally considered more favorable, as it indicates that the extract is more potent and requires a lower concentration to inhibit a biological process by 50%. In simpler terms, a lower IC_50_ means the substance is more effective at its intended purpose. In our study, the impact on Caco-2 cells was consistent with previously reported findings, and there was no significant difference between the ozonized and non-ozonized extracts. This outcome aligns with the results reported by Hazekawa et al. (2019) [51]. For Wi-38 cells, which represent normal cells, a higher IC_50_ value is generally considered advantageous, as it indicates that a greater concentration of the tested compound is required to inhibit the growth of normal cells by 50% compared to cancer cells. Such a higher IC_50_ for normal cells suggests greater selectivity toward cancer cells and reduced cytotoxicity to healthy cells. This observation agrees with the findings of Berrouet et al. (2020) [52]. Statistical analysis revealed a significant difference between the ozonized and non-ozonized extracts in their effects on normal cells, with the ozonized extract demonstrating a more favorable safety profile. Thus, while ozonation did not alter the extract’s anticancer potency, it shifted its action toward greater safety for normal cells. In other words, ozonation enhanced the extract’s safety profile while maintaining its anticancer efficacy, supporting the in vitro hypothesis regarding the benefits of ozonized extracts.

## 3. Materials and Methods

### 3.1. Collection and Extraction of O. basilicum L.

Fresh leaves of *O. basilicum* L. (Saudi basil) were collected from the campus of the College of Applied Medical Sciences, University of Ha’il, Hail, Saudi Arabia. The plant was authenticated at the College of Science, Jazan University, Jazan, Saudi Arabia. The leaves were shade-dried at 30 °C, ground into a fine powder, and stored in airtight containers at 25 °C. Five grams of powdered leaves were soaked in 50 mL of 85% methanol in a sealed glass container and left at room temperature (24 °C) for 24 h. The mixture was then sonicated for 60 min at 40 °C and filtered. The filtration was concentrated using a rotary evaporator under vacuum at 40 °C, and the resulting extract, that concentrated using the rotary evaporator under vacuum at 40 °C, was stored at 4 °C until use.

### 3.2. HPLC Analysis

Phytochemical profiling of non-ozonated and ozonated *O. basilicum* extracts was performed using an HPLC system (Waters Corporation, Milford, MA, USA) coupled to a TOF-MS detector with electrospray ionization in negative mode. Separation was carried out on a BEH Shield RP18 column (1.7 μm, 2.1 × 100 mm; Waters Corporation, Milford, MA, USA) using a gradient of acetonitrile (mobile phase B) and water with 1% acetic acid (mobile phase A) as described by Verni et al. [53]. Data were analyzed using MassLynx 4.1 software relative to standard mixture of phenolic compounds.

### 3.3. In Vitro α-Amylase Inhibition Assay

The α-amylase inhibitory activity was determined using the 3,5-dinitrosalicylic acid (DNSA) method [54]. Extracts (1.9–1000 μg/mL) were prepared by dissolving in 10% DMSO, followed by dilution in phosphate buffer (0.02 M Na_2_HPO_4_, 0.006 M NaCl, pH 6.9). A 200 μL aliquot of the extract was mixed with 200 μL of α-amylase solution (2 U/mL) and incubated at 30 °C for 10 min. Subsequently, 200 μL of starch solution was added and incubated for 3 min. The reaction was stopped by adding 200 μL of DNSA reagent (12 g sodium potassium tartrate tetrahydrate, 8 mL 2 M NaOH, and 20 mL 96 mM DNSA solution) and heating at 85–90 °C for 10 min. After cooling, 5 mL of distilled water was added, and the absorbance was read at 540 nm using a Biosystem 310 UV–Vis spectrophotometer (PerkinElmer, Shelton, CT, USA). Blanks were prepared without the enzyme. The percentage of inhibition was calculated as follows:

$$\alpha -amylase\;inhibition\;(\%)\;=\;\frac{Abs.\;100\%\;\left(control\right)\;-\;Abs.\;sample}{Abs.\;100\%\;(control)}\times 100$$

IC_50_ values (concentration required to inhibit 50% of the enzyme) were determined by plotting inhibition against extract concentration relative to the acarbose standard.

### 3.4. In Vitro α-Glucosidase Inhibition Assay

The α-glucosidase inhibitory activity was assessed using a modified Pistia and Hollingsworth method [55]. Extracts (1.97–1000 μg/mL, 50 μL) were incubated at 37 °C for 20 min with 10 μL of α-glucosidase (1 U/mL; Sigma, St. Louis, MO, USA) and 125 μL of phosphate buffer (0.1 M, pH 6.8). The reaction was initiated by adding 20 μL of 1 M p-nitrophenyl-α-D-glucopyranoside (pNPG) and incubating for 30 min. Afterward, 50 μL of 0.1 N Na_2_CO_3_ was added to stop the reaction, and absorbance was measured at 405 nm. Blanks were prepared without enzyme. Inhibition percentage was calculated as follows:

$$\alpha \;-\;glucosidase\;inhibition\;(\%)=\frac{100-(Abs.\;blank\;-\;Abs.\;sample)}{Abs.\;blank}\times 100$$

IC_50_ values (concentration required to inhibit 50% of the enzyme) were determined by plotting inhibition against extract concentration.

### 3.5. In Vitro Anti-Alzheimer Activity (Butyrylcholinesterase Inhibition)

Butyrylcholinesterase (BChE) inhibitory activity was evaluated using butyrylthiocholine iodide as a substrate and 5,5′-dithiobis-(2-nitrobenzoic acid) (DTNB) as a chromogenic reagent [56,57]. BChE stock solution (3.47 U/mL) was prepared in 20 mM sodium phosphate buffer (pH 7.6). Plant extracts were prepared in the same buffer (100 μg/mL). The reaction mixture contained 10 μL of the test sample, 79 μL of buffer, and 1 μL of enzyme, incubated for 15 min at 37 °C. Subsequently, 10 μL of 4 mM butyrylthiocholine iodide was added and incubated for 30 min. The reaction was terminated by adding 900 μL of DTNB-phosphate-ethanol reagent, and absorbance was recorded at 405 nm. IC_50_ values were determined from dose–response inhibition curve relative to acarbose standard [58].

### 3.6. In Vitro Anti-Inflammatory Activity (Hypotonic Hemolysis Method)

#### 3.6.1. Preparation of Red Blood Cell (RBC) Suspension

Venous blood (3 mL) was collected from a healthy volunteer into heparinized tubes (ethical approval obtained; date 3 August 2024). Samples were centrifuged at 3000× *g* for 10 min, and red blood cells (RBCs) were washed with an equal volume of normal saline. The packed RBCs were reconstituted as a 40% *v*/*v* suspension in isotonic buffer (10 mM phosphate buffer, 0.2 g NaH_2_PO_4_, 1.15 g Na_2_HPO_4_, 9 g NaCl per liter, pH 7.4), sodium dodecyl sulfate (SDS) was applied to induce 100% of hemolysis [59].

#### 3.6.2. Hypotonic Hemolysis Assay

Extracts (100–1000 μg/mL) were added to hypotonic and isotonic solutions (5 mL each). Control tubes contained distilled water and indomethacin. RBC suspension (0.1 mL) was added to each tube, incubated at room temperature for 1 h, and centrifuged at 1300× *g* for 3 min. The absorbance of the supernatant was measured at 540 nm. The percentage inhibition of hemolysis was calculated as follows [60]:

$$Inhibition\;of\;haemolysis\;(\%)=\frac{1-(Abs.2-Abs.1)}{Abs.3-Abs.1}\times 100$$

where A_1_, A_2_, and A_3_ represent absorbances of test sample in isotonic solution, test sample in hypotonic solution, and control in hypotonic solution, respectively.

### 3.7. The Determination of Anti-Helicobacter pylori Activity

#### 3.7.1. Qualitative Agar Diffusion Assay

A clinical strain of *H. pylori* was obtained from Ain Shams Specialized Hospital, identified by VITEK^®^ 2 (Salt Lake City, UT, USA) and confirmed by VITEK^®^ MS (Salt Lake City, UT, USA). The bacterial suspension (1.0 × 10^8^ CFU/mL) was prepared to a McFarland standard of 2 [61]. Mueller–Hinton agar plates with 10% sheep blood were inoculated, and wells (6–8 mm) were filled with 100 μL of plant extract. DMSO served as negative control; amoxicillin, clarithromycin, and metronidazole served as positive controls. Plates were incubated under microaerophilic conditions (37 °C, 72 h) in a specialized anaerobic jar containing a gas mixture of 5–10% oxygen, 5–10% hydrogen, and 5–10% carbon dioxide, and the inhibition zones were measured [62].

#### 3.7.2. Quantitative Hemolysin Inhibition Assay

Hemolysin inhibition was performed as described by Rossignol et al. [63]. Supernatants from MIC-treated or untreated *H. pylori* cultures were incubated with 2% RBC suspension at 37 °C for 2 h, and absorbance was read at 540 nm. Hemolysis (%) was calculated as follows:

$$Hemolysis(\%)=\frac{Sample\;with\;bacterial\;isolate-Negative\;control}{Positive\;control-Negative\;control}\times 100$$

### 3.8. MIC and MBC Determination

Minimum inhibitory concentrations (MICs) were determined using a microdilution technique with Mueller–Hinton broth supplemented with horse blood [64]. Serial dilutions of extracts (0.98–1000 μg/mL) were prepared in 96-well plates, inoculated with *H. pylori* (3 × 10^6^ CFU/mL), and incubated at 35 °C for 72 h under 15% CO_2_. MIC was defined as the lowest concentration that completely inhibited growth. Minimum bactericidal concentration (MBC) was determined by subculturing from wells with no visible growth onto blood agar plates and incubating for 72 h. The MBC/MIC ratio was calculated to determine bactericidal versus bacteriostatic effects [65].

### 3.9. Cytotoxicity Assay

WI-38 (normal human lung fibroblasts) and Caco-2 (human colorectal adenocarcinoma) cells were cultured in RPMI-1640 medium supplemented with 10% fetal bovine serum (FBS), 100 U/mL penicillin, and 100 μg/mL streptomycin under standard conditions (37 °C, 5% CO_2_, humidified atmosphere). For the assay, cells were seeded into 96-well flat-bottom plates at a density of 1 × 10^5^ cells/mL (100 μL/well) and incubated for 24 h to allow monolayer formation. The culture medium was then removed and replaced with serial dilutions of the test extracts prepared in RPMI medium containing 2% FBS. Vehicle controls (2% FBS RPMI medium without extract) and positive controls (doxorubicin) were included in each experiment. After 24 h of treatment, cell morphology was examined under an inverted microscope for signs of cytotoxicity. Cell viability was quantified using the MTT assay [55,56,57,58]: 20 μL of MTT solution (5 mg/mL in PBS) was added to each well, and plates were incubated for 4 h at 37 °C. The resulting formazan crystals were solubilized by adding 200 μL of DMSO per well and gently shaking for 10 min. Absorbance was measured at 560 nm with background correction at 620 nm using a microplate reader. IC_50_ values were calculated using non-linear regression analysis.

### 3.10. Ozone Generation

Ozone was produced using a dielectric barrier discharge plasma reactor (DBDPR) consisting of two electrodes separated by a dielectric barrier [66,67]. The generated ozone was bubbled into a bacterial suspension (6 × 10^8^ CFU/mL) at pH 6–8 and 20–25 °C for 10–40 min (1.2 mg/L/h). Samples were incubated at 37 °C for 30 min to allow ozone consumption, followed by addition of resazurin dye (20 μL) and further incubation for 4 h [68,69].

### 3.11. Preparation of Resazurin Dye

Resazurin (0.34 g) was dissolved in 50 mL of sterile distilled water with continuous mixing for 1 h, protected from light, and stored in dark bottles. Reduction of resazurin (blue) to resorufin (pink/red) was measured at 570 nm and 600 nm [70].

### 3.12. Statistical Analysis

All experiments were performed in triplicate. Data are expressed as mean ± standard deviation (SD) or mean ± standard error (SE). Statistical analyses were conducted using SPSS version 25.0 and Microsoft Excel 365. One-way analysis of variance (ANOVA) followed by Tukey’s post hoc test was applied, with a significance threshold of *p* < 0.05.

## 4. Conclusions

This study provides comprehensive evidence that ozonation significantly modulates the phytochemical profile of *O. basilicum* L. and enhances its biological activities. Compared to non-ozonated (untreated) Saudi basil extracts, ozonated methanolic extracts demonstrated superior antidiabetic effects through potent inhibition of carbohydrate-hydrolyzing enzymes, exhibited enhanced BChE inhibition with potential neuroprotective relevance, and produced stronger anti-inflammatory activity, as evidenced by RBC membrane stabilization. Furthermore, ozonation markedly improved antibacterial efficacy against *H. pylori* while reducing cytotoxicity toward normal human fibroblasts and intestinal epithelial cells, thereby enhancing the safety profile. These preliminary findings highlight the potential of ozonated *O. basilicum* as a safe and effective natural therapeutic candidate for metabolic, neurodegenerative, and infectious disorders. The study has several limitations, including the scalability of ozonation for pharmaceutical production and the potential degradation of bioactive compounds during the process. Future in vivo experiments, pharmacokinetic studies, and well-designed clinical trials are warranted to confirm the safety and efficacy of these findings and to explore their translational potential in pharmaceutical applications.

## Figures and Tables

**Table 1:** Determination of the concentrations of the components of non-ozonated Saudi basil (untreated) and ozonated Saudi basil compared to the standard material (Different letters above the numbers indicate a significant difference where *p* ≤ 0.05).

Components	Standard		Non-Ozonated Saudi Basil		Ozonated Saudi Basil	
	Conc. (µg/mL)	Area	Conc. (µg/mL)	Area	Conc. (µg/mL)	Area
Gallic acid	20	248.33	727.67 ^a^	58.61	723.78 ^a^	58.29
Chlorogenic acid	50	391.71	382.63 ^a^	48.84	1207.83 ^b^	154.17
Catechin	75	329.15	0.00 ^a^	0.00	63.59 ^b^	14.49
Methyl gallate	15	291.59	125.02 ^a^	6.43	101.77 ^a^	5.24
Caffeic acid	18	220.63	288.91 ^a^	23.57	203.97 ^a^	16.64
Syringic acid	17.2	289.68	141.54 ^a^	8.40	179.96 ^a^	10.69
Rutin	50	410.85	65.71 ^a^	8.00	40.53 ^a^	4.93
Ellagic acid	70	568.31	115.05 ^a^	14.17	75.46 ^a^	9.30
Coumaric acid	20	600.85	122.46 ^a^	4.08	81.18 ^a^	2.70
Vanillin	12.9	350.04	769.67 ^a^	28.36	473.89 ^a^	17.46
Ferulic acid	20	357.33	625.38 ^a^	35.00	415.99 ^a^	23.28
Naringenin	30	334.36	39.51	3.55	15.32	1.38
Rosmarinic acid	50	501.19	1604.89 ^a^	160.11	1015.34 ^a^	101.29
Daidzein	20	319.64	692.21 ^a^	43.31	408.94 ^a^	25.59
Quercetin	20	300.41	1590.07 ^a^	105.86	965.95 ^a^	64.31
Cinnamic acid	10	584.22	212.01 ^a^	3.63	131.64 ^a^	2.25
Kaempferol	20	279.09	810.83 ^a^	58.10	504.15 ^a^	36.13
Hesperetin	20	418.68	23.65 ^a^	1.13	15.19 ^a^	0.73

**Table 2:** In vitro α-amylase inhibitor assay using standard acarbose and non-ozonated (untreated) and ozonated Saudi basil as test substance.

Conc. (µg/mL)	Mean A_540_			α-Amylase Inhibition (%)			SD			SE		
	Acar	SB	OSB	Acar	SB	OSB	Acar	SB	OSB	Acar	SB	OSB
1000	0.053	0.093	0.072	95.8	92.5	94.2	0.002	0.004	0.003	0.001	0.001	0.001
500	0.092	0.136	0.101	92.6	89.1	91.9	0.004	0.003	0.002	0.001	0.001	0.001
250	0.127	0.196	0.146	89.8	84.3	88.3	0.004	0.003	0.002	0.001	0.001	0.000
125	0.153	0.282	0.177	87.7	77.4	85.8	0.003	0.003	0.006	0.001	0.001	0.002
62.5	0.237	0.392	0.266	81.0	68.5	78.7	0.004	0.004	0.004	0.001	0.001	0.001
31.25	0.323	0.480	0.359	74.1	61.5	71.2	0.003	0.003	0.007	0.001	0.001	0.002
15.62	0.419	0.589	0.487	66.4	52.7	60.9	0.007	0.003	0.014	0.002	0.001	0.005
7.81	0.522	0.708	0.581	58.1	43.2	53.4	0.005	0.006	0.009	0.001	0.002	0.003
3.9	0.615	0.816	0.678	50.7	34.5	45.7	0.003	0.003	0.003	0.001	0.001	0.001
1.95	0.782	0.918	0.786	37.3	26.4	36.9	0.003	0.003	0.002	0.001	0.001	0.001
100%	1.247	1.247	1.247	0.00	0.00	0.00	0.003	0.003	0.003	0.001	0.001	0.001
IC_50_				3.47 ^a^	13.6 ^b^	5.09 ^a^						

Acar: acarbose; SB: non-ozonated Saudi basil; OSB: ozonated Saudi basil; SD: standard deviation; SE: standard error. Different letters above IC_50_ values indicate significant differences (*p* ≤ 0.05) as determined by one-way ANOVA.

**Table 3:** In vitro α-glucosidase inhibitor assay using standard acarbose and non-ozonated (untreated) and ozonated Saudi basil as test substance.

Conc. (µg/mL)	Mean A_540_			α-Glucosidase Inhibition (%)			SD			SE		
	Acar	SB	OSB	Acar	SB	OSB	Acar	SB	OSB	Acar	SB	OSB
1000	0.049	0.160	0.123	97.2	90.9	93.0	0.002	0.003	0.004	0.001	0.001	0.001
500.00	0.095	0.279	0.226	94.7	84.3	87.3	0.004	0.002	0.008	0.001	0.000	0.002
250.00	0.180	0.368	0.340	89.9	79.3	80.9	0.002	0.003	0.004	0.000	0.001	0.001
125.00	0.275	0.483	0.430	84.6	72.8	75.9	0.007	0.002	0.007	0.002	0.001	0.002
62.50	0.393	0.588	0.537	77.9	67.0	69.8	0.003	0.003	0.003	0.001	0.001	0.001
31.25	0.506	0.705	0.647	71.6	60.4	63.7	0.005	0.003	0.006	0.002	0.001	0.002
15.63	0.620	0.815	0.737	65.2	54.2	58.6	0.004	0.003	0.004	0.001	0.001	0.001
7.81	0.746	0.927	0.840	58.1	47.9	52.8	0.004	0.004	0.004	0.001	0.001	0.001
3.91	0.862	1.032	0.942	51.6	42.0	47.1	0.003	0.003	0.004	0.001	0.001	0.001
1.95	0.973	1.126	1.096	45.3	36.7	38.4	0.005	0.004	0.008	0.002	0.001	0.003
Control	1.745	1.745	1.745	0.00	0.00	0.00	0.006	0.006	0.006	0.002	0.002	0.002
IC_50_	2.91 ^a^	9.42 ^b^	6.15 ^b^									

Acar; acarbose, SB; non-ozonated Saudi basil, OSB; ozonated Saudi basil, SD; standard deviation, SE; standard error (Different letters above IC_50_ values indicate significant differences (*p* ≤ 0.05) as determined by one-way ANOVA).

**Table 4:** Anti-Alzheimer assay using standard rivastigmine and non-ozonated (untreated) and ozonated Saudi basil as test substance.

Conc. (µg/mL)	Mean A_405_			BChE Inhibition (%)			SD			SE		
	RIV	SB	OSB	RIV	SB	OSB	RIV	SB	OSB	RIV	SB	OSB
100	0.009	0.080	0.053	95.6	62.1	74.8	0.001	0.001	0.001	0.000	0.000	0.000
50	0.020	0.096	0.068	90.5	54.4	67.8	0.001	0.001	0.001	0.000	0.000	0.000
25	0.028	0.107	0.101	86.7	49.4	52.0	0.001	0.001	0.001	0.000	0.000	0.000
12.5	0.044	0.118	0.111	79.2	44.3	47.6	0.002	0.002	0.002	0.000	0.000	0.000
6.25	0.054	0.145	0.124	74.4	31.4	41.2	0.002	0.002	0.002	0.000	0.000	0.000
3.125	0.070	0.158	0.138	66.9	25.2	34.8	0.002	0.002	0.002	0.001	0.001	0.001
1.56	0.081	0.181	0.159	61.7	14.5	24.8	0.004	0.004	0.004	0.001	0.001	0.001
0.78	0.093	0.193	0.175	55.8	8.7	17.2	0.002	0.002	0.002	0.001	0.001	0.001
0.39	0.108	0.200	0.188	48.9	5.3	11.0	0.003	0.003	0.003	0.001	0.001	0.001
0.195	0.127	0.201	0.197	39.9	4.9	6.6	0.003	0.003	0.003	0.001	0.001	0.001
0.000	0.2113	0.2113	0.2113	0.00	0.00	0.00	0.000	0.000	0.000	0.000	0.000	0.000
IC_50_				0.46 ^a^	31.8 ^a^	13.4 ^c^						

RIV; rivastigmine, BChE; butyryl cholinesterase, SB; non-ozonated Saudi basil, OSB; ozonated Saudi basil, SD; standard deviation, SE; standard error (Different letters above IC_50_ values indicate significant differences (*p* ≤ 0.05) as determined by one-way ANOVA).

**Table 5:** Anti-inflammatory assay using standard indomethacin and non-ozonated (untreated) and ozonated Saudi basil as the test substance.

Conc. (µg/mL)	Mean A_540_						Hemolysis Inhibition (%)			SD			SE		
	Hypotonic Sol.			Isotonic Sol.											
	IND	SB	OSB	IND	SB	OSB	IND	SB	OSB	IND	SB	OSB	IND	SB	OSB
Control	1.184	1.184	1.184	0.001	0.001	0.001	0.00	0.00	0.00	0.003	0.003	0.003	0.001	0.001	0.001
1000	0.035	0.062	0.055	0.021	0.021	0.021	98.8	96.5	97.0	0.003	0.005	0.005	0.001	0.001	0.002
500	0.083	0.102	0.094	0.015	0.015	0.015	94.2	92.6	93.2	0.002	0.003	0.002	0.001	0.001	0.001
250	0.104	0.141	0.138	0.011	0.011	0.011	92.0	88.9	89.2	0.003	0.008	0.005	0.001	0.003	0.001
125	0.181	0.215	0.207	0.009	0.009	0.009	85.4	82.4	83.1	0.005	0.003	0.005	0.002	0.001	0.001
62.5	0.262	0.292	0.288	0.005	0.005	0.005	78.2	75.6	76.0	0.006	0.003	0.003	0.002	0.001	0.001
31.25	0.345	0.396	0.391	0.003	0.003	0.003	71.0	66.8	67.2	0.003	0.004	0.003	0.001	0.001	0.001
15.62	0.449	0.507	0.502	0.002	0.002	0.002	62.2	57.2	57.7	0.007	0.003	0.005	0.002	0.001	0.001
7.8	0.562	0.626	0.621	0.001	0.001	0.001	52.6	47.1	47.6	0.003	0.003	0.002	0.001	0.001	0.001
3.9	0.623	0.737	0.728	0.001	0.001	0.001	47.4	37.8	38.5	0.004	0.004	0.001	0.001	0.001	0.000
IC50	4.41 ^a^	8.44 ^b^	8.04 ^b^												

IND; indomethacin, SB; non-ozonated Saudi basil, OSB; ozonated Saudi basil, SD; standard deviation, SE; standard error (Different letters above IC_50_ values indicate significant differences (*p* ≤ 0.05) as determined by one-way ANOVA).

**Table 6:** Qualitative assay of Anti-*Helicobacter pylori* activity of non-ozonated (untreated) and ozonated Saudi basil.

Substance	Anti-*Helicobacter pylori* Activity (mm)				SD	SE
	R 1	R 2	R 3	Mean		
Omeclamox-Pak (control)	24	25	24	24.5	0.577	0.190
Non-ozonated Saudi basil	19	18	19	18.7	0.577	0.188
Ozonated Saudi basil	26	27	27	26.7	0.577	0.188

SD; standard deviation, SE; standard error.

**Table 7:** Inhibition of hemolysis (%) caused by *Helicobacter pylori* of non-ozonated (untreated) and ozonated Saudi basil.

Sample	A_540_ of Sample with RBCs		A_540_ of Sample with Isotonic Solution		Hemolysis (%)		SD		SE	
	SB	OSB	SB	OSB	SB	OSB	SB	OSB	SB	OSB
Control	1.735	1.735	0.0001	0.0001	100	100	0.011	0.011	0.004	0.004
(25% MIC)	0.417	0.212	0.008	0.007	23.6 ^a^	11.8 ^b^	0.006	0.003	0.002	0.001
(50% MIC)	0.108	0.106	0.004	0.003	6.0 ^a^	6.0 ^a^	0.002	0.006	0.001	0.002
(75% MIC)	0.059	0.042	0.000	0.000	3.4 ^a^	2.4 ^a^	0.004	0.003	0.001	0.001

SB; non-ozonated Saudi basil, OSB; ozonated Saudi basil, SD; standard deviation, SE; standard error (Different letters above sub-MIC levels (25%, 50%, and 75% MIC) indicate significant differences (*p* ≤ 0.05) as determined by *t*-test analysis).

**Table 8:** MIC and MBC of non-ozonated (untreated) and ozonated Saudi basil against *Helicobacter pylori*.

Sample	MIC (µg/mL)	MBC (µg/mL)	MBC/MIC Index (µg/mL)
Omeclamox-Pak (Control)	31.25	62.5	2 *
Non-ozonated Saudi basil	15.62	31.25	2 *
Ozonated Saudi basil	31.25	31.25	1 *

*n* * ≤ 4 indicates bactericidal activity, *n* > 4 indicates bacteriostatic activity.

**Table 9:** Cytotoxicity of non-ozonated (untreated) and ozonated Saudi basil on WI-38 cell line (*t*-test comparisons of the IC_50_ values for both treatments indicate significant differences where *p* ≤ 0.05).

Sample	Conc. (µg/mL)	Mean of A_560_	Viability (%)	Toxicity (%)	SD	SE	IC_50_ (µg/mL)
WI-38 cell line	None	0.719	100	0.00	None	0.001	None
Non-ozonated Saudi basil	1000	0.06	8.35	91.65	4.01	0.004	191.06 ± 4.01 ^a^
	500	0.085	11.87	88.13	4.01	0.002	
	250	0.232	32.32	67.68	4.01	0.007	
	125	0.483	67.23	32.77	4.01	0.010	
	62.5	0.694	96.57	3.43	4.01	0.003	
	31.25	0.719	100.0	0.00	4.01	0.001	
Ozonated Saudi basil	1000	0.035	4.96	95.04	4.84	0.002	437.89 ± 4.84 ^b^
	500	0.275	38.2	61.75	4.84	0.005	
	250	0.643	89.5	10.52	4.84	0.005	
	125	0.705	98.05	1.947	4.84	0.003	
	62.5	0.718	99.9	0.092	4.84	0.0008	
	31.25	0.719	100	0.00	4.84	0.0005	

SD; standard deviation, SE; standard error. Different letters (a:b) indicate a significant difference at *p* ≤ 0.05.

**Table 10:** Cytotoxicity of non-ozonated (untreated) and ozonated Saudi basil on Caco-2 cell line (*t*-test comparisons of the IC_50_ values for both treatments indicate no significant differences where *p* > 0.05).

Sample	Conc. (µg/mL)	Mean of A_560_	Viability (%)	Toxicity (%)	SD	SE	IC_50_ µg/mL
Caco-2 cell line	None	0.665	100	0.00	None	0.003	None
Non-ozonated Saudi basil	1000	0.017	2.656	97.34	1.16	0.0003	103.70 ± 1.16 ^a^
	500	0.018	2.756	97.24	1.16	0.0003	
	250	0.026	3.959	96.04	1.16	0.0035	
	125	0.250	37.64	62.35	1.16	0.0077	
	62.5	0.487	73.23	26.76	1.16	0.0066	
	31.25	0.650	97.74	2.255	1.16	0.0046	
Ozonated Saudi basil	1000	0.018	2.757	97.24	0.82	0.0003	149.14 ± 0.8 ^a^
	500	0.018	2.807	97.19	0.82	0.0008	
	250	0.020	3.057	96.94	0.82	0.0008	
	125	0.349	52.48	47.52	0.82	0.0040	
	62.5	0.662	99.55	0.451	0.82	0.0020	
	31.25	0.665	100	0.00	0.82	0.0015	

SD; standard deviation, SE; standard error. Values sharing the same superscript letter are not significantly different (*p* ≥ 0.05)

## Data Availability

Data that supports the findings of this study are available within the article and from the corresponding author upon request.
